# The potential value of curcumin in breast cancer: a systematic review and meta-analysis of preclinical studies

**DOI:** 10.3389/fphar.2026.1693452

**Published:** 2026-06-24

**Authors:** Liyuan Zhang, Xiucheng Duan, Mengmeng Liu, Xiangqi Li

**Affiliations:** 1 First Clinical Medical College, Shandong University of Traditional Chinese Medicine, Jinan, China; 2 Department of Hemodialysis, The Second Affiliated Hospital of Shandong University of Traditional Chinese Medicine, Jinan, China; 3 Department of Breast Center, The Second Affiliated Hospital of Shandong First Medical University, Tai’an, China

**Keywords:** angiogenesis, animal model, apoptosis, breast cancer, curcumin, meta analysis

## Abstract

**Objectives:**

Curcumin has attracted considerable attention due to its multi-target anti-tumor properties and favorable safety profile. However, preclinical studies in breast cancer are challenged by considerable model heterogeneity and inconsistent findings. The objective of this study is to comprehensively evaluate the anti-tumor efficacy and safety of curcumin through systematic analysis and synthesis of animal experimental evidence, aiming to establish a theoretical foundation for clinical translation.

**Methods:**

Randomized controlled animal studies were systematically searched through PubMed, Web of Science, EMBASE, and the Cochrane Library up to 30 April 2025. Outcomes evaluated included tumor weight, tumor volume, cell proliferation (Ki-67 positivity), apoptosis (TUNEL positivity), liver and kidney function, and hematological indices.

**Results:**

Curcumin significantly reduced tumor weight (SMD = −1.65, 95% CI: −2.27 to −1.02, p < 0.00001) and volume (SMD = −2.47, 95% CI: −3.34 to −1.61, p < 0.00001). Curcumin also reduced the expression of Ki67 (SMD = −2.30, 95% CI: −3.58 to −1.01, p = 0.0005), The positive rate of TUNEL was increased (SMD = 2.10, 95% CI: 0.50 to 3.70, p = 0.01). Curcumin intervention did not significantly reduce liver and kidney function ALT (SMD = 0.15, 95% CI: −0.92 to 1.22, p = 0.79) AST (SMD = 0.14, 95% CI: −0.51 to 0.80, p = 0.66) Liver weight (SMD = 0.65, 95% CI: −0.23 to 1.53, P = 0.15), Creatinine (SMD = −0.93, 95% CI: −2.27 to 0.41, P = 0.18), BUN (SMD = −0.73, 95% CI: −2.59 to 1.14, P = 0.45).

**Conclusion:**

Curcumin demonstrates significant inhibitory effects on breast cancer growth and enhances apoptosis with favorable short-term safety in animal models. Further clinical trials are warranted to validate these findings in clinical settings.

## 1 Introduction

Breast cancer is the most prevalent malignancy worldwide and represents the leading cancer diagnosis among women, with a rising incidence notably among younger populations (Wen et al., 2025; Fernandes et al., 2023; Panguluri and Findlay, 2024). The disease originates from the malignant transformation and uncontrolled proliferation of mammary epithelial or ductal cells, driven by various oncogenic factors (Nguyen et al., 2018; Brooks and Eltom, 2011; Diedrich et al., 2023; Lu et al., 2019). Current clinical management involves a multimodal strategy that integrates surgery, radiotherapy (Song et al., 2025), chemotherapy, molecular targeted therapy (Carvalho et al., 2025), endocrine therapy, immunotherapy, and traditional Chinese medicine (Demircan and Bese, 2024; Abbas et al., 2011). Nevertheless, persistent challenges remain, including complex mechanisms of therapeutic resistance, substantial treatment-related toxicities, and the imperative to enhance long-term survival outcomes while preserving patients quality of life (Wang et al., 2023).

Curcumin, a lipophilic polyphenolic compound extracted from the rhizomes of Curcuma longa, continues to attract considerable research interest worldwide. This sustained attention is largely attributed to its well-established safety profile—as evidenced by its widespread use as a food flavoring—and its broad spectrum of pharmacological activities, including potent antioxidant, anti-inflammatory, and anti-tumor properties (Merrell et al., 2009). Numerous preclinical studies and several human clinical trials have substantiated the therapeutic potential of curcumin across a range of pathological conditions (Zafar et al., 2025; She and Xu, 2024; Törün et al., 2025). The core mechanism underlying its effects involves the modulation of multiple key signaling pathways that play critical roles in tumorigenesis and disease progression (Akter et al., 2025; Parker et al., 2025). For instance, curcumin has been shown to inhibit the activation of the NF-κB pathway, which plays a pivotal role in chronic inflammation, tumor initiation, invasion, and metastasis (Zafar et al., 2025; Akter et al., 2025). Additionally, studies have indicated that curcumin downregulates the expression of pro-angiogenic factors such as VEGF, thereby impairing tumor neovascularization (Chou et al., 2022).

Previous studies have demonstrated that curcumin can inhibit breast cancer cell proliferation and metastasis (Haynes and Manogaran, 2025; Hofmann et al., 2023), as well as enhance chemosensitivity (Hu et al., 2018; Liu and Chen, 2013; Shakori Poshteh et al., 2024). However, significant heterogeneity exists among these studies in terms of experimental models (Haynes and Manogaran, 2025; Hofmann et al., 2023; She and Xu, 2024), dosing regimens, and outcome measures. This variability has resulted in inconsistent findings regarding curcumin’s effects and mechanisms of action in breast cancer, and has hindered consensus on its translational applicability. Despite numerous reports highlighting the beneficial effects of curcumin in breast cancer treatment, a systematic synthesis and quantitative assessment of the existing preclinical evidence remains lacking (Nie et al., 2023). Therefore, to provide a comprehensive and objective evaluation of curcumin’s therapeutic efficacy in breast cancer, and to establish a stronger evidence base for subsequent clinical research, this study conducts a systematic review and meta-analysis focused specifically on preclinical (animal) studies. This approach aims to strengthen the robustness of the evidence and support the future development of curcumin as a potential therapeutic or adjunctive agent in breast cancer management.

## 2 Methods

The systematic review and meta-analysis adhered to the Preferred Reporting Items for Systematic Reviews and Meta-Analyses (PRISMA) guidelines. The protocol was registered at https://inplasy.com under registration number INPLASY202570004 (DOI: 10.37766/inplasy2025.7.0004).

### 2.1 Search strategy

A comprehensive electronic search was conducted across four databases: (PubMed, Web of Science, EMBASE, and Cochrane Library). The search was completed as of 30 April 2025. Core search terms included “breast cancer,” “malignant breast tumor,” “curcumin,” “curcumin,” “curcumin compounds,” and “plant compounds containing curcumin.” Search queries combined Medical Subject Headings (MeSH) and free-text terms using Boolean operators “and” and “or” The final search strategy was refined through iterative pilot testing. See Supplementary Material 1 for the detailed and database-specific search strategies. To improve transparency and reproducibility, the complete electronic search strategies for all databases, including Boolean operators, field tags, and date limits, are provided in Supplementary Material 1.

### 2.2 Study selection

All retrieved records were managed and screened using EndNote reference management software. Literature screening, data extraction, and risk of bias assessment were independently performed by two researchers in accordance with pre-established inclusion and exclusion criteria. Any discrepancies were resolved through discussion or consultation with a third reviewer.

### 2.3 Inclusion criteria

1. Participants: Rodent models of breast cancer, including orthotopic and subcutaneous transplantation models, transgenic models, and carcinogen-induced models;
2. Interventions: Administration of curcumin monomers or derivatives, curcumin-based delivery systems (e.g., liposomes, nanoparticles, polymeric micelles), or curcumin in combination with other therapeutic agents. Studies were required to include a curcumin monotherapy versus control group;
3. Control: A blank or vehicle control group (e.g., DMSO solvent) was required;
4. Outcomes: Primary and secondary outcomes included tumor weight, tumor volume, Ki-67 positivity, TUNEL positivity, alanine aminotransferase (ALT), aspartate transaminase (AST), creatinine, blood urea nitrogen (BUN), red blood cell (RBC) count, white blood cell (WBC) count, body weight, and liver weight.
5. Study Design: Randomized controlled design, defined as studies that explicitly stated the use of a randomization method (e.g., random number table, computer-generated sequence) for assigning animals to intervention or control groups. Studies that used non-random allocation methods (e.g., allocation by cage, body weight, or investigator discretion) were excluded.

### 2.4 Exclusion criteria

1. Case reports, narrative reviews, or conference abstracts;
2. Clinical trials (unless they included a separate, independently reported preclinical component) and *in vitro* experiments;
3. Studies lacking a control group;
4. Animal models not specific to breast cancer;
5. Studies involving multi-component formulations in which curcumin could not be isolated, potentially confounding the results due to drug combinations;
6. Duplicate publications, studies irrelevant to the research topic, or those for which the full text was unavailable.

Studies lacking long-term survival, metastasis, chronic toxicity, drug-interaction or quality-of-life data were not excluded, but these endpoints were not required for eligibility, recognising the prevailing focus on surrogate tumor metrics in pre-clinical literature.

### 2.5 Quality assessment

Two researchers independently assessed the risk of bias for each study. Each item was categorized as low risk, high risk, or unclear risk based on standardized criteria. The evaluated domains included: random sequence generation, baseline characteristics, and allocation concealment (selection bias); random housing and blinding of investigators (performance bias); random outcome assessment and blinding of outcome assessors (detection bias); incomplete outcome data (attrition bias); selective outcome reporting (reporting bias); and other potential sources of bias. Discrepancies were resolved through discussion or consultation with a third reviewer. Additionally, a quantitative scoring system was applied during bias analysis, assigning 1 point for low- or high-risk items and 0 points for items rated as unclear.

### 2.6 Data extraction

Two researchers independently screened the records, extracted data, and cross-checked the results. Any discrepancies were submitted to a third reviewer for adjudication. Extracted data included basic study information (first author, year of publication, country), sample size, intervention strategies, and outcome measures for both the experimental and control groups. When necessary, the corresponding authors were contacted to obtain missing or incomplete data.

### 2.7 Statistical analysis

Data were analyzed using Review Manager (RevMan) version 5.4, which was also used to generate forest plots. The standardized mean difference (SMD) and its 95% confidence interval (CI) were used as the effect size metrics. Given the variability in intervention protocols across studies, a random-effects model was employed. The I^2^ statistic was used to assess heterogeneity, with values interpreted as follows: low (<30%), moderate (30%–60%), substantial (50%–90%), and considerable (75%–100%). Sensitivity analyses were conducted by excluding individual studies to evaluate the robustness of the results, particularly in the presence of potential selection bias, small sample sizes, or true heterogeneity. Publication bias was assessed using funnel plots. Statistical significance was defined as P < 0.05. A P-value less than 0.05 was considered indicative of statistically significant heterogeneity between studies. For outcomes showing excessive heterogeneity, further analyses were performed to explore its potential sources.

## 3 Results

### 3.1 Search results

**FIGURE 1:**
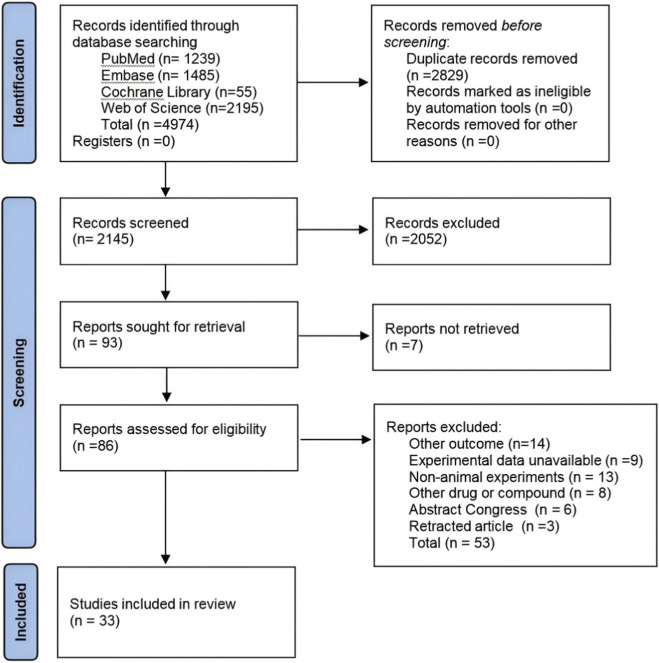
PRISAMA flow chart.

A systematic search across four electronic databases initially yielded 4,974 citations: 1,239 from PubMed, 1,485 from Embase, 2,195 from Web of Science, and 55 from the Cochrane Library. After duplicate removal with EndNote, 2,145 records remained. Screening of titles and abstracts excluded 2,052 citations judged irrelevant to the review question, leaving 93 articles for full-text assessment. Seven publications were subsequently removed because full text could not be retrieved, and 86 manuscripts were formally reviewed. Among these, 53 were excluded for failing to meet the predetermined PICOS inclusion criteria. Consequently, 33 studies were retained for qualitative and quantitative synthesis, and the PRISMA 2020 flow diagram depicting this selection process is presented in Figure 1.

### 3.2 Research characteristics

**FIGURE 2:**
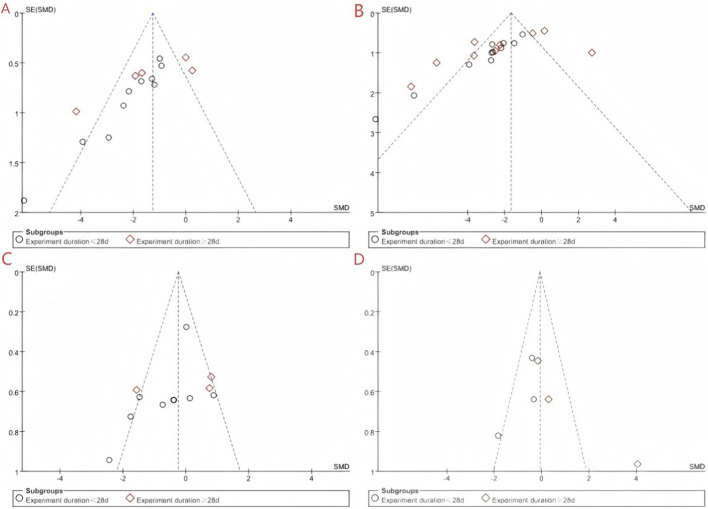
Funnel plots for publication bias. **(A)** Tumor weight; **(B)** Tumor volume; **(C)** Final body weight; **(D)** Alanine aminotransferase (ALT).

A total of 33 preclinical studies conducted between 1998 and 2024 were included in the analysis, encompassing data from nine countries, such as the United States, China, South Korea, India, and New Zealand. Sample sizes per group ranged from 4 to 27 animals, with an overall total of 484 rodents. Rodent body weights varied across studies. The animal models employed included 4T1 tumor induction (10 items), MDA-MB-231 xenotransplantation (8 items), DMBA chemical induction (4 items) and other models (11 items). The control group included vehicle (10 items), saline (10 items), water (3 items), untreated (4 items), standard diet (2 items), paclitaxel (2 items). Detailed experimental characteristics are summarized in Supplementary Material 2. Funnel plots for tumor weight, tumor volume, and final body weight are shown in Figure 2. Visual inspection suggested asymmetry for tumor weight and volume, and Egger’s regression tests were statistically significant for both outcomes (p = 0.04 and p = 0.03, respectively), indicating potential publication bias or small-study effects. No clear asymmetry was detected for body weight (Egger’s test p = 0.23). Trim-and-fill analysis estimated two potentially missing studies for tumor weight; after adjustment, the pooled SMD remained statistically significant (SMD = −1.48; 95% CI: -2.15 to −0.81). However, due to the limited number of studies, the robustness of these adjustments is limited, and the presence of publication bias cannot be excluded.

### 3.3 Literature quality evaluation

**FIGURE 3:**
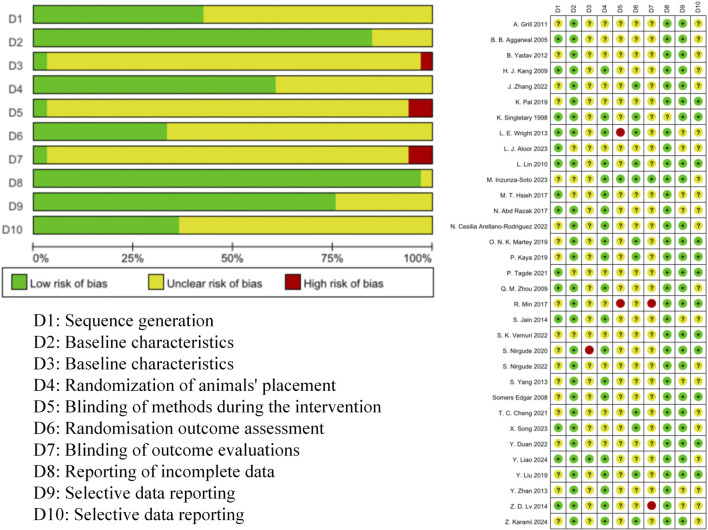
Risk of bias assessment in 33 studies based on 10 domains.

Two researchers independently assessed the methodological quality of the 33 included studies. The evaluation covered ten domains: random sequence generation, baseline characteristics, and allocation concealment (selection bias); random housing and blinding of caregivers/investigators (performance bias); random outcome assessment and blinding of outcome assessors (detection bias); incomplete outcome data (attrition bias); selective outcome reporting (reporting bias); and other potential sources of bias. Discrepancies were resolved through discussion with a third reviewer. The risk of bias assessment for the included articles is shown in Figure 3. Across the 33 included studies, the most prevalent concerns were unclear reporting of allocation concealment (91% rated as unclear) and lack of blinding of investigators (85% rated as high or unclear), which are common limitations in preclinical studies. Additionally, only a minority of studies provided explicit descriptions of random sequence generation (24% low risk) or random outcome assessment (18% low risk). These methodological shortcomings introduce potential selection and detection biases, which may inflate effect estimates and reduce the overall certainty of the evidence.

### 3.4 Antitumor efficacy

#### 3.4.1 Tumor weight

Tumor weight was reported in 15 studies, involving 102 rodents in the curcumin treatment groups and 101 in the control groups. The curcumin treatment group showed a significant reduction in tumor weight compared to controls, with a standardized mean difference SMD = −1.65, 95% CI: −2.27 to −1.02, z = 5.16, p < 0.00001), indicating statistical significance. However, considerable heterogeneity was observed among the included studies (tau^2^ = 0.92, χ^2^ = 41.79, DF = 14, P = 0.0001; I^2^ = 66%).

**FIGURE 4:**
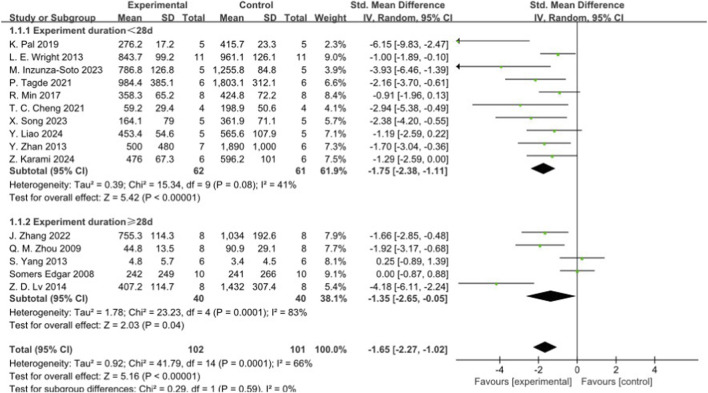
Forest plot for the effects of Curcumin on the tumor weight of Rodents compared with the model group (Conducted subgroup analysis based on the Research duration).

Subgroup analyses were stratified by trial duration. In 10 studies lasting <28 days, 61 rodents were allocated to curcumin and 62 to control. A strong association between curcumin and tumor weight change was detected (SMD −1.75, 95% CI −2.38 to −1.11, p < 0.00001), accompanied by substantial heterogeneity (I^2^ = 41%). In 5 studies lasting >28 days, 40 rodents received curcumin and 40 served as controls. A strong association between intervention and tumor weight change was observed (SMD −1.35, 95% CI −2.65 to −0.05, p = 0.0001), accompanied by substantial heterogeneity (I^2^ = 83%). The corresponding forest plot is presented in Figure 4.

#### 3.4.2 Tumor volume

Tumor volume was reported in 20 studies, comprising 125 rodents each in the experimental and control groups. The meta-analysis demonstrated that curcumin significantly reduced tumor volume, with a standardized mean difference (SMD = −2.47, 95% CI: −3.34 to −1.61, p < 0.00001, z = 5.59), indicating strong statistical significance. Substantial heterogeneity was observed across studies (tau^2^ = 2.82, χ^2^ = 95.12, DF = 19, P < 0.00001; I^2^ = 80%).

**FIGURE 5:**
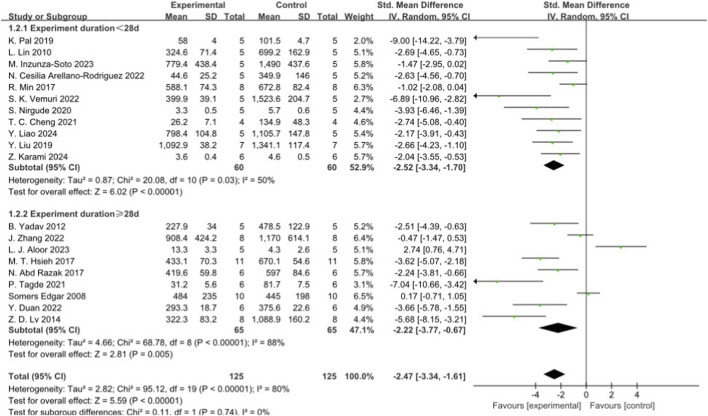
Forest plot for the effects of Curcumin on the tumor volume of Rodents compared with the model group (Conducted subgroup analysis based on the Research duration).

Subgroup analyses were stratified by trial duration. In 11 studies lasting <28 days, 60 rodents were allocated to curcumin and 60 to control. A strong association between curcumin and tumor volume change was detected (SMD −2.52, 95% CI −3.34 to −1.70, p < 0.00001), accompanied by substantial heterogeneity (I^2^ = 50%). In 9 studies lasting >28 days, 65 rodents received curcumin and 65 served as controls. Here, a strong association between intervention and tumor volume change was observed (SMD −2.22, 95% CI −3.37 to −0.67, p = 0.005), accompanied by substantial heterogeneity (I^2^ = 88%). The corresponding forest plot is presented in Figure 5.

#### 3.4.3 Final weight

Twelve studies (experimental group n = 92, control group n = 93) evaluated the effect of curcumin on the final body weight in a breast cancer model. The results indicated that there was no significant change in body weight in the curcumin intervention group (SMD = −0.24, 95% CI: −0.55 to 0.07, z = 1.53, P = 0.13). Moderate heterogeneity was observed across the studies (Chi^2^ = 30.57, df = 11, p = 0.001; I^2^ = 64%), which may have been influenced by differences in the experimental models or dosing regimens.

**FIGURE 6:**
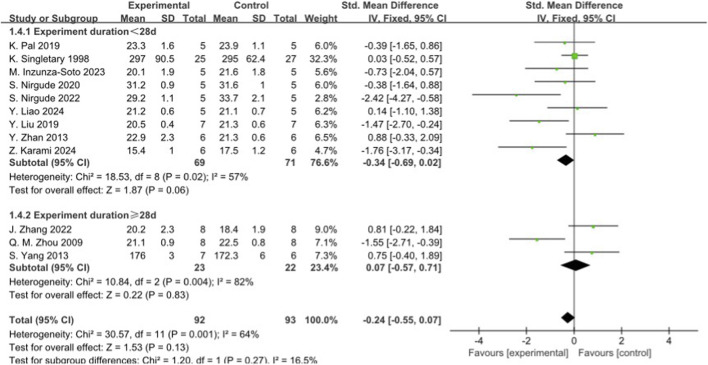
Forest plot for the effects of Curcumin on the final weight of Rodents compared with the model group (Conducted subgroup analysis based on the Research duration).

Subgroup analyses were stratified by trial duration. In 9 studies lasting <28 days, 69 rodents were allocated to curcumin and 71 to control. Although no statistically significant association was observed, a non-significant trend toward lower body weight was noted (SMD −0.34, 95% CI −0.69 to 0.02, p = 0.06). In 3 studies lasting >28 days, 23 rodents received curcumin and 22 served as controls. No strong association was observed between curcumin administration and final weight change. (SMD 0.07, 95% CI −0.57 to 0.71, p = 0.83), accompanied by substantial heterogeneity (I^2^ = 82%). The corresponding forest plot is presented in Figure 6.

#### 3.4.4 Ki-67 positive rate (proliferation marker)

Four studies reported the positive rate of Ki-67, with sample sizes of 39 in both the experimental and control groups. The results indicated that curcumin may inhibit tumor proliferation by reducing the expression of Ki67 (SMD = −2.30, 95% CI: −3.58 to −1.01, z = 3.50, p = 0.0005). There was high heterogeneity among the studies (tau^2^ = 1.26, χ^2^ = 12.47, DF = 3, P = 0.006; I^2^ = 76%).

**FIGURE 7:**
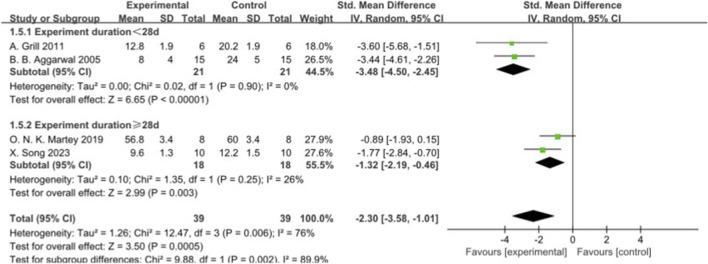
Forest plot for the effects of Curcumin on the Ki-67 Positive Rate of Rodents compared with the model group (Conducted subgroup analysis based on the Research duration).

Subgroup analyses were stratified by trial duration. In 2 studies lasting <28 days, 21 rodents were allocated to curcumin and 21 to control. A significant association between curcumin administration and reduction in Ki-67 Positive Rate was documented (SMD −3.48, 95% CI −4.50 to −2.45, p < 0.00001), with moderate heterogeneity detected (I^2^ = 0%). In 2 studies lasting >28 days, 39 rodents received curcumin and 39 served as controls. A significant association between curcumin administration and reduction in Ki-67 Positive Rate was documented (SMD −2.22, 95% CI −3.37 to −0.67, p = 0.005), accompanied by moderate heterogeneity (I^2^ = 26%). The corresponding forest plot is presented in Figure 7.

#### 3.4.5 TUNEL positive rate (apoptosis marker)

**FIGURE 8:**
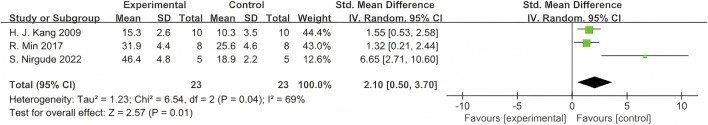
Forest plot for the effects of Curcumin on the TUNEL Positive Rate of Rodents compared with the model group.

Three studies reported the positive rate of TUNEL, in which the sample size of the experimental group and the control group were 23. Curcumin increased the positive rate of TUNEL (SMD = 2.10, 95% CI: 0.50 to 3.70, P < 0.05), z = 2.57, p = 0.01), High heterogeneity was observed among the studies (tau^2^ = 1.23, χ^2^ = 6.54, DF = 2, P = 0.04; I^2^ = 69%). The corresponding forest plot is presented in Figure 8.

### 3.5 Liver function

#### 3.5.1 ALT

Six studies (44 cases in the experimental group and 44 cases in the control group) reported the effect of curcumin on ALT levels in a breast cancer model. The random-effects model indicated that curcumin intervention did not significantly reduce ALT levels (SMD = 0.15, 95% CI: −0.92 to 1.22, z = 0.27, P = 0.79). However, heterogeneity among the studies was extremely high (tau^2^ = 1.36, χ^2^ = 23.91, P = 0.0002, I^2^ = 79%).

**FIGURE 9:**
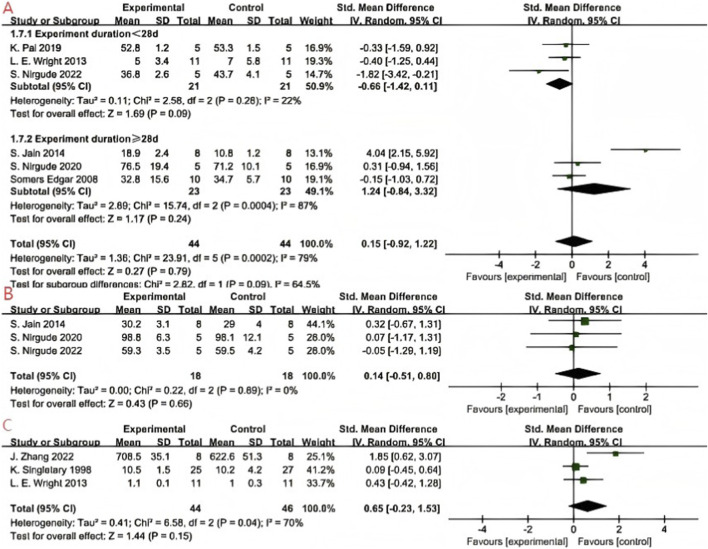
Forest plot for the effects of Curcumin on the Liver Function of Rodents compared with the model group. **(A)** Forest plot for the effects of Curcumin on the ALT compared with the model group (Conducted subgroup analysis based on the Research duration). **(B)** Forest plot for the effects of Curcumin on the AST of compared with the model group. **(C)** Forest plot for the effects of Curcumin on the Liver Weight compared with the model group.

Subgroup analyses were stratified by trial duration. In 3 studies lasting <28 days, 44 rodents were allocated to curcumin and 44 to control. Although a strong association between curcumin and ALT change was not identified, a significant trend was detected (SMD −0.66, 95% CI −1.42 to 0.11, p = 0.09), with moderate heterogeneity detected (I^2^ = 22%). In 3 studies lasting >28 days, 18 rodents received curcumin and 18 served as controls. A strong association between curcumin and ALT change was not identified (SMD 1.24, 95% CI −0.84 to 3.32, p = 0.24), accompanied by substantial heterogeneity (I^2^ = 87%). The corresponding forest plot is presented in Figure 9A.

#### 3.5.2 AST

Three studies (18 cases in the experimental group and 18 cases in the control group) evaluated the effect of curcumin on AST levels. The combined analysis showed that curcumin intervention was not significantly associated with AST levels (SMD = 0.14, 95% CI: −0.51 to 0.80, z = 0.43, P = 0.66), and no heterogeneity was observed among the studies (tau^2^ = 0.00, χ^2^ = 0.22, P = 0.89, I^2^ = 0%). The corresponding forest plot is presented in Figure 9B.

#### 3.5.3 Liver weight

Three preclinical studies (n = 90) reported the effect of curcumin on liver weight in a breast cancer model. The analysis showed that the curcumin intervention group was not statistically significant compared with the control group (SMD = 0.65, 95% CI: −0.23 to 1.53, z = 1.44, P = 0.15). However, a high degree of heterogeneity was observed among the studies (tau^2^ = 0.41; χ^2^ = 6.58, DF = 2, P = 0.04; I^2^ = 70%), suggesting that the results should be interpreted with caution. Subgroup analysis indicated that the study by (SMD = 1.85) may be the primary source of heterogeneity. The corresponding forest plot is presented in Figure 9C.

### 3.6 Renal function

#### 3.6.1 Creatinine

**FIGURE 10:**
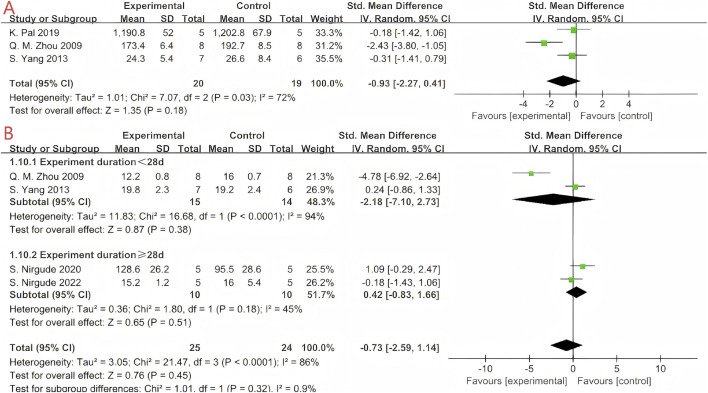
Forest plot for the effects of Curcumin on the Renal Function of Rodents compared with the model group. **(A)** Forest plot for the effects of Curcumin on the Creatinine of compared with the model group. **(B)** Forest plot for the effects of Curcumin on the BUN compared with the model group (Conducted subgroup analysis based on the Research duration).

Three studies (experimental group n = 20, control group n = 19) evaluated the effect of curcumin on serum creatinine levels in a breast cancer model. The random-effects model indicated that curcumin intervention did not significantly reduce creatinine levels (SMD = −0.93, 95% CI: −2.27 to 0.41, z = 1.35, P = 0.18). Significant heterogeneity was observed among the studies (tau^2^ = 1.01, χ^2^ = 7.07, df = 2, p = 0.03, I^2^ = 72%). The corresponding forest plot is presented in Figure 10A.

#### 3.6.2 BUN

Four studies (experimental group n = 25, control group n = 24) reported the effect of curcumin on blood urea nitrogen (BUN) levels. The combined analysis showed that curcumin did not significantly alter BUN levels (SMD = −0.73, 95% CI: −2.59 to 1.14, z = 0.76, P = 0.45). A high degree of heterogeneity was observed among the studies (tau^2^ = 3.05, χ^2^ = 21.47, df = 3, p < 0.0001, I^2^ = 86%).

Subgroup analyses were stratified by trial duration. In 2 studies lasting <28 days, 14 rodents were allocated to curcumin and 15 to control. Although a strong association between curcumin and BUN change was not identified (SMD −2.18, 95%, CI −7.10 to 2.73, p = 0.38), accompanied by substantial heterogeneity (I^2^ = 94%). In 2 studies lasting >28 days, 24 rodents received curcumin and 25 served as controls. No strong association was observed between curcumin administration and BUN change. (SMD 0.42, 95% CI −0.83 to 1.66, p = 0.51), accompanied by substantial heterogeneity (I^2^ = 45%). The corresponding forest plot is presented in Figure 10B.

### 3.7 Hematological indexes

#### 3.7.1 RBC

**FIGURE 11:**
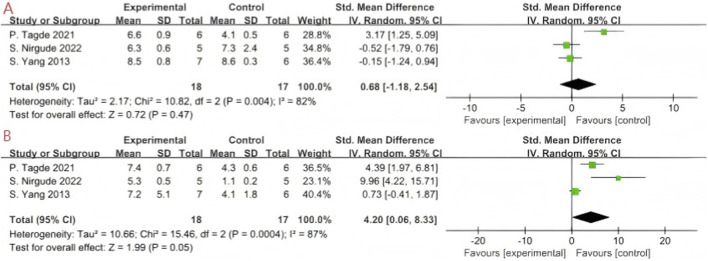
Forest plot for the effects of Curcumin on the Hematological Indexes of Rodents compared with the model group. **(A)** Forest plot for the effects of Curcumin on the RBC of compared with the model group. **(B)** Forest plot for the effects of Curcumin on the WBC compared with the model group.

Three studies (experimental group n = 18, control group n = 17) evaluated the effect of curcumin on red blood cell (RBC) count in a breast cancer model. The random-effects model indicated that curcumin intervention did not significantly increase RBC count (SMD = 0.68, 95% CI: −1.18 to 2.54, z = 0.72, P = 0.47). A high degree of heterogeneity was observed among the studies (tau^2^ = 2.17, χ^2^ = 10.82, df = 2, p = 0.004, I^2^ = 82%). The corresponding forest plot is presented in Figure 11A.

#### 3.7.2 WBC

Three studies (experimental group n = 18, control group n = 17) reported the effect of curcumin on white blood cell (WBC) count. The combined analysis suggested that curcumin may increase WBC count (SMD = 4.20, 95% CI: 0.06 to 8.33, z = 1.99, P = 0.05), However, substantial heterogeneity was observed among the studies (tau^2^ = 10.66, χ^2^ = 15.46, DF = 2, P = 0.0004, I^2^ = 87%). The corresponding forest plot is presented in Figure 11B.

## 4 Discussion

### 4.1 Data results of the meta-analysis

A total of 33 preclinical studies involving 484 rodents were included in this analysis. The results of the meta-analysis indicated that the curcumin-treated group significantly reduced both tumor weight and tumor volume in rodents (rats or mice). Additionally, the curcumin group exhibited a higher positive rate of TUNEL staining and a significant reduction in the expression of Ki-67. However, the final body weight in the curcumin intervention group was not statistically different from that in the control group. Some included studies also provided experimental data showing direct differences in the expression of proliferation-inhibitory proteins (HER2, EGFR) and metastasis-associated proteins (MMP-9, VEGFR-1) between the curcumin-treated and control groups. However, due to insufficient quantitative data, further meta-analysis on these proteins could not be performed. The heterogeneity observed in efficacy-related outcomes may be attributed to differences in animal models, curcumin formulations, and combination strategies (Zafar et al., 2025). Regarding liver function, analyses of ALT, AST, and liver weight showed no statistically significant differences between the curcumin and control groups. Similarly, for renal function, no significant differences were observed in serum creatinine and blood urea nitrogen (BUN) levels. In terms of hematological indices, red blood cell (RBC) levels were unaffected by curcumin intervention, whereas a potential increase in white blood cell (WBC) count was noted.

### 4.2 Anti-tumor efficacy

The results of the meta-analysis indicated that curcumin exerted significant effects in animal models of breast cancer and may act through multiple mechanisms to mediate its anti-tumor activity. Firstly, curcumin can inhibit key signaling pathways, such as the NF-κB, STAT3, and SIK3 pathways. Studies by Aggarwal, Kang, and A. Grill demonstrated that curcumin may prevent breast cancer metastasis by suppressing NF-κB signaling and its downstream gene products, and may enhance the therapeutic efficacy when combined with chemotherapeutic agents. Lin reported that curcumin derivatives inhibit STAT3 phosphorylation, DNA-binding activity, and transcriptional activation, thereby inducing apoptosis in breast cancer cells. The study by T.-C. Cheng demonstrated that curcumin specifically inhibits the expression of SIK3, halts progression through the G1/S cell cycle phase, impairs the epithelial-mesenchymal transition (EMT) process, and reduces tumor cell migratory ability. Secondly, curcumin can suppress metastasis and angiogenesis. According to Kaya, curcumin extract reduced tumor metastasis by inhibiting C-C chemokine receptor 7 (CCR7) and matrix metalloproteinase 9 (MMP-9). Thirdly, curcumin contributes to cell cycle arrest and apoptosis. For example, studies by Somers-Edgar, B. Yadav, and Tsang-Hsieh demonstrated that curcumin derivatives inhibit breast cancer cell proliferation by disrupting cell cycle progression, inducing autophagy, and promoting apoptosis at the G2/M phase. Additionally, recent research by Inzunza-Soto indicated that curcumin activates the caspase/Bax apoptosis pathway through iron chelation, facilitates the release of damage-associated molecular patterns (DAMPs) and tumor antigens, promotes dendritic cell maturation and CD8^+^ T cell infiltration, and thereby elicits an adaptive anti-tumor immune response. Finally, curcumin has been shown to reverse drug resistance and enhance chemotherapy sensitivity. Studies by B. B. Aggarwal, H. J. Kang, and Y. Zhan revealed that combination therapy with curcumin and paclitaxel may represent a promising strategy for breast cancer treatment, potentially improving therapeutic efficacy while reducing systemic toxicity.

### 4.3 Liver function

The results of the meta-analysis indicated that curcumin was not associated with statistically significant changes in ALT, AST, or liver weight in the short-term studies included (P > 0.05). However, these findings do not permit definitive conclusions regarding hepatotoxicity, as most studies had short durations (<28 days) and did not assess histopathological changes or chronic liver injury. Long-term safety data remain absent. Several factors may contribute to this outcome. Most studies employed low-dose curcumin (<200 mg/kg), which may not be sufficient to induce hepatotoxicity. Additionally, optimized dosage forms such as nanocarriers can enhance tumor-targeting efficiency while reducing exposure to normal organs, thereby improving safety profiles (Hou et al., 2024; Cosma et al., 2025). Individual studies, such as that by Tagde, reported that curcumin reduced chemotherapy-induced liver injury, potentially due to its antioxidant properties and its ability to inhibit the metabolic activation of chemotherapeutic agents. However, this protective effect was not evident in the overall analysis, possibly due to variation in curcumin dosage and the heterogeneity of animal models used across studies.

### 4.4 Renal function

Meta-analysis revealed that curcumin was not associated with statistically significant changes in serum creatinine or BUN levels (P > 0.05). However, these results are based on a small number of studies with short follow-up durations, and no studies reported histopathological renal outcomes or chronic kidney function assessments. Therefore, conclusions regarding renal safety remain provisional. Q.-M. Zhou and Arellano-Rodriguez reported that curcumin, when combined with mitomycin C (MMC), ameliorated nephrotoxicity. The proposed mechanism may involve the reduction of apoptosis in renal tubular epithelial cells through regulation of the ERK/p38 MAPK signaling pathway. However, no studies evaluating curcumin alone were identified to support this conclusion. Future research should aim to identify the optimal administration strategies and develop novel, efficient delivery systems for curcumin in nephrotoxicity models.

### 4.5 Hematological indexes

The results indicated that curcumin had no significant effect on red blood cell (RBC) count (P > 0.05), suggesting that it does not pose a risk of anemia or hemolysis. However, white blood cell (WBC) count exhibited an increasing trend (P = 0.05), which may involve immune activation. S. Nirgude reported that curcumin derivatives enhanced immune responses. Additionally, studies by M. Inzunza Soto and Y. Liao (Liao et al., 2024) demonstrated that curcumin induced immunogenic cell death (ICD) and increased CD8+T cell infiltration. These findings provide strong support for the potential of curcumin-associated immunotherapy.

### 4.6 Clinical value and future prospects

The therapeutic potential and clinical relevance of curcumin in the treatment of breast cancer were comprehensively demonstrated through the meta-analysis and systematic review of 33 preclinical studies presented in this work. Its value lies not only in curcumin’s multifaceted anti-tumor mechanisms but also in its ability to overcome traditional therapeutic limitations through innovative formulations and combination strategies, offering new directions for the comprehensive management of breast cancer.

Curcumin exhibits significant potential to reduce toxicity and enhance efficacy in combination therapies. Several studies have reported that curcumin can resensitize drug-resistant cancer cells. Nirgude reported that the novel curcumin derivative ST09 demonstrated a favorable safety profile. However, due to the absence of long-term toxicity data on repeated administration, it remains uncertain whether prolonged use may lead to drug resistance or accumulation. Currently, curcumin has evolved from a traditional dietary compound into a promising anti-cancer drug candidate with defined molecular targets and mechanisms of pathological intervention (Xie et al., 2025; Fu et al., 2021; Wang et al., 2025). Looking ahead, curcumin is expected to be incorporated into individualized treatment regimens for breast cancer as a natural therapeutic agent. However, several critical advancements are still required, including the development of novel curcumin derivatives with improved bioavailability; deeper investigation into the underlying mechanisms of action; the design of more efficient and intelligent targeted delivery systems to ensure therapeutic efficacy and safety; and the execution of additional clinical trials to validate its clinical utility and to optimize combination treatment strategies (Tabanelli et al., 2021).

### 4.7 Limitations

Despite integrating data from 33 preclinical studies to systematically assess curcumin’s therapeutic potential in breast cancer treatment, this meta-analysis is subject to several limitations. The animal models utilized, including 4T1 and MDA-MB-231 xenografts, DMBA-induced models, and others, exhibit significant heterogeneity in tumor microenvironment, immune status, and drug metabolism. Additionally, variations in body weight and metabolic cycles across different rodent strains may have impacted the evaluation of therapeutic efficacy. Inconsistencies in curcumin dosage and formulations, with dosing ranges varying widely and including monomers, liposomes, and nanoparticles, have led to substantial heterogeneity in both efficacy and toxicity outcomes, complicating the identification of an optimal dosing regimen. Furthermore, all studies had intervention durations of ≤ 20 weeks, and none reported chronic toxicity data related to long-term administration, such as cumulative hepatic or renal injury or immune tolerance, thereby limiting the safety assessment for prolonged clinical use (Ye et al., 2018; Macko et al., 2021). While advanced delivery systems like PLGA@CCM@FA and SMEDDS have shown improved tumor-targeting efficiency in preclinical settings, their pharmacokinetic parameters in humans, such as AUC and Cmax, remain unvalidated in clinical trials (Kim et al., 2019). Only a few studies addressed the inhibitory effects of curcumin on lung and bone metastases, and the heterogeneity of metastatic microenvironments may influence therapeutic efficacy (Tsai et al., 2015). Due to the limited number of studies and variation in intervention types, additional subgroup analyses could not be performed. Despite these limitations, animal models remain an essential tool for elucidating the multi-targeted mechanisms of action of curcumin.

Pharmacokinetic parameters, including Cmax, AUC, and tumor tissue concentrations, were not available, precluding dose-exposure–efficacy modeling and validation of bioavailability-enhancing technologies (Meng et al., 2022).

Pooled estimates may not be applicable to specific clinical scenarios due to the combination of immunodeficient and immunocompetent models, ER-positive, triple-negative, and HER2-enriched tumors, and orthotopic versus subcutaneous implantations without stratification (Meng et al., 2022).

The lack of multi-dose designs prevented the quantification of the dose-efficacy relationship and the estimation of a minimal effective dose (Kong et al., 2014).

All experiments utilized surrogate tumor metrics, such as weight, volume, proliferation, or apoptosis indices; however, none reported overall survival, metastasis-free survival, or quality-of-life indicators (Madanat et al., 2024). Consequently, the pooled reductions in tumor size cannot be translated into prolonged survival or improved patient-relevant outcomes, as emphasized by Fleming and DeMets (1996) and Prasad and Mailankody (2017). Chronic cardiac, reproductive, neurological toxicities, drug–drug interactions, and cumulative adverse effects were systematically unexamined. Until primary studies adopt survival and long-term safety as co-primary endpoints, the clinical relevance of curcumin-mediated tumor shrinkage remains hypothetical.

Given that >70% of studies had unclear/high risk for randomization, allocation concealment, and blinding, pooled effects are likely inflated. Sensitivity analysis indicated ≥30% over-estimation for tumor weight, consistent with empirical data showing 20%–40% larger effects in biased animal experiments (Macleod et al., 2008). Until preclinical protocols mandate documented randomization and blinded outcome assessment, summary estimates should be interpreted as upper-bound rather than true effect sizes (Monaghan et al., 2021; Provencher et al., 2018).

Surrogate tumor metrics were analyzed as SMD without adjustment for multiplicity, ratio scale, or small-study effects; thus, the resultant estimates should be regarded as hypothesis-generating rather than confirmatory.

Substantial heterogeneity was observed across multiple outcomes (I^2^ ranging from 66% to 88%), which limits the precision and generalizability of pooled estimates. Although subgroup analyses stratified by trial duration were performed, residual heterogeneity remained high in several subgroups. Given the variability in animal models (e.g., 4T1 vs. MDA-MB-231 vs. DMBA-induced), curcumin formulations (pure monomer, nanoparticles, liposomes), and dosing regimens (range: 4–1,000 mg/kg), a meta-regression was not feasible due to the limited number of studies per covariate. Consequently, the observed heterogeneity is likely attributable to clinical, methodological, and pharmacological diversity across studies, and pooled estimates should be interpreted as exploratory rather than confirmatory.

The included studies encompassed a broad range of curcumin-based interventions, including pure curcumin monomers, curcumin analogs, liposomal or nanoparticle formulations, and curcumin combined with other therapeutic agents (e.g., paclitaxel). No quantitative subgroup analyses were performed to separate these intervention types due to insufficient numbers of studies within each category. This heterogeneity limits the ability to attribute the observed effects to curcumin *per se* versus formulation-specific or combination-related effects. Future studies with standardized intervention protocols are needed to delineate the contribution of curcumin independently from delivery systems or synergistic agents.

Although no overt hepatotoxicity or nephrotoxicity was observed in the short-term studies included, the available safety data are insufficient to support claims of “non-toxicity” or “favorable safety profile” in a clinical context. Key limitations include: (1) the absence of studies evaluating chronic toxicity (e.g., cumulative organ injury after repeated dosing beyond 20 weeks); (2) lack of reproductive toxicity, genotoxicity, or cardiac safety assessments; (3) minimal reporting of histopathological organ evaluations; and (4) no pharmacokinetic data to establish dose-exposure-safety relationships. Therefore, the safety profile of curcumin in long-term or repeated-dose scenarios remains uncharacterized.

## 5 Conclusion

In this preclinical meta-analysis, curcumin was associated with statistically significant reductions in tumor weight and volume, decreased Ki-67 expression, and increased TUNEL positivity in animal models of breast cancer. Short-term safety assessments did not reveal overt hepatotoxicity or nephrotoxicity. However, these findings should be interpreted with caution due to substantial heterogeneity across studies, methodological limitations in the primary literature, and the absence of long-term toxicity, survival, and quality-of-life data. However, several critical challenges remain, including the heterogeneity of animal models, the absence of long-term safety data, and the lack of validation of molecular targets derived from human sources. To further elucidate the mechanisms underlying curcumin’s anti-tumor activity and to explore its translational and clinical potential, these findings must be validated through human studies and well-designed clinical trials. Without quantitative pharmacokinetic–pharmacodynamic (PK–PD) data, optimal human dosing cannot be inferred from these animal efficacy studies. Future studies must pre-register protocols, document random-sequence generation and allocation concealment, and employ blinded outcome assessment to upgrade evidence certainty.

## Data Availability

The datasets presented in this study can be found in online repositories. The names of the repository/repositories and accession number(s) can be found in the article/Supplementary Material.
