# Supplementary material for: The potential value of curcumin in breast cancer: a systematic review and meta-analysis of preclinical studies
Source: Front Pharmacol. 2026 Jun 24;17:1693452. doi: 10.3389/fphar.2026.1693452 (PMC13341899; doi:10.3389/fphar.2026.1693452)
Supplement: Supplementary file 1 [file Supplementaryfile1.pdf]

Supplementary Material 1： Search strategy on each database.

Database	Search Strategy
**Pubmed and Cochrane Library**	#1 Breast Neoplasms[MeSH Terms]<br>#2 ((((((((((((((((((((((((((((((((((Neoplasm, Breast) OR (Neoplasms, Breast)) OR (Breast Tumors)) OR (Breast Tumor)) OR (Tumor, Breast)) OR (Tumors, Breast)) OR (Breast Cancer)) OR (Cancer, Breast)) OR (Cancer of Breast)) OR (Cancer of the Breast)) OR (Malignant Neoplasm of Breast)) OR (Breast Malignant Neoplasm)) OR (Breast Malignant Neoplasms)) OR (Malignant Tumor of Breast)) OR (Breast Malignant Tumor)) OR (Breast Malignant Tumors)) OR (Mammary Cancer)) OR (Cancer, Mammary)) OR (Cancers, Mammary)) OR (Mammary Cancers)) OR (Mammary Neoplasms, Human)) OR (Human Mammary Neoplasm)) OR (Human Mammary Neoplasms)) OR (Neoplasm, Human Mammary)) OR (Neoplasms, Human Mammary)) OR (Mammary Neoplasm, Human)) OR (Breast Carcinoma)) OR (Breast Carcinomas)) OR (Carcinoma, Breast)) OR (Carcinomas, Breast)) OR (Carcinoma, Human Mammary)) OR (Carcinomas, Human Mammary)) OR (Human Mammary Carcinomas)) OR (Mammary Carcinomas, Human)) OR (Human Mammary Carcinoma)<br>#3 (#1) OR (#2)<br>#4 (Curcumin[MeSH Terms]) OR (Curcuma[MeSH Terms])<br>#5 (((((((((((((((((((((Curcumin) OR (Curcuma)) OR (Curcumin Phytosome)) OR (Phytosome, Curcumin)) OR (Diferuloylmethane)) OR (Turmeric Yellow)) OR (Yellow, Turmeric)) OR (Mervia)) OR (Curcumas)) OR (Curcuma longa)) OR (Curcuma longas)) OR (longa, Curcuma)) OR (Tumeric)) OR (Tumerics)) OR (Turmeric)) OR (Turmerics)) OR (Curcuma zedoaria)) OR (Curcuma zedoarias)) OR (zedoaria, Curcuma)) OR (Zedoary zedoaria)) OR (zedoaria, Zedoary)) OR (Zedoary zedoarias)<br>#6 (#4) OR (#5)<br>#7 (#3) AND (#6)
**Web of Science**	#1 ((((((((((((((((((((((((((((((((((((AB=(Breast Neoplasms)) OR AB=(Neoplasm, Breast)) OR AB=(Neoplasms, Breast)) OR AB=(Breast Tumors)) OR AB=(Breast Tumor)) OR AB=(Tumor, Breast)) OR AB=(Tumors, Breast)) OR AB=(Breast Cancer)) OR AB=(Cancer, Breast)) OR AB=(Cancer of Breast)) OR AB=(Cancer of the Breast)) OR AB=(Malignant Neoplasm of Breast)) OR AB=(Breast Malignant Neoplasm)) OR AB=(Breast Malignant Neoplasms)) OR AB=(Malignant Tumor of Breast)) OR AB=(Breast Malignant Tumor)) OR AB=(Breast Malignant Tumors)) OR AB=(Mammary Cancer)) OR AB=(Cancer, Mammary)) OR AB=(Cancers, Mammary)) OR AB=(Mammary Cancers)) OR AB=(Mammary Neoplasms, Human)) OR AB=(Human Mammary Neoplasm)) OR AB=(Human Mammary Neoplasms)) OR AB=(Neoplasm, Human Mammary)) OR AB=(Neoplasms, Human Mammary)) OR AB=(Mammary Neoplasm, Human)) OR AB=(Breast Carcinoma)) OR AB=(Breast Carcinomas)) OR AB=(Carcinoma, Breast)) OR AB=(Carcinomas, Breast)) OR AB=(Carcinoma, Human Mammary)) OR AB=(Carcinomas, Human Mammary)) OR AB=(Human Mammary Carcinomas)) OR AB=(Mammary Carcinomas, Human)) OR AB=(Human Mammary Carcinoma))<br>#2 ((((((((((((((((((((((AB=(Curcumin)) OR AB=(Curcuma)) OR AB=(Curcumin Phytosome)) OR AB=(Phytosome, Curcumin)) OR AB=(Diferuloylmethane)) OR AB=(Turmeric Yellow)) OR AB=(Yellow, Turmeric)) OR AB=(Mervia)) OR AB=(Curcumas)) OR AB=(Curcuma longa)) OR AB=(Curcuma longas)) OR AB=(longa, Curcuma)) OR AB=(Tumeric)) OR AB=(Tumerics)) OR AB=(Turmeric)) OR AB=(Turmerics)) OR AB=(Curcuma zedoaria)) OR AB=(Curcuma zedoarias)) OR AB=(zedoaria, Curcuma)) OR AB=(Zedoary zedoaria)) OR AB=(zedoaria, Zedoary)) OR AB=(Zedoary zedoarias))<br>#3 (#1) AND (#2)
**Embase**	#1 'breast neoplasms':ab,ti OR 'neoplasm, breast':ab,ti OR 'neoplasms, breast':ab,ti OR 'breast tumors':ab,ti OR 'breast tumor':ab,ti OR 'tumor, breast':ab,ti OR 'tumors, breast':ab,ti OR 'breast cancer':ab,ti OR 'cancer, breast':ab,ti OR 'cancer of breast':ab,ti OR 'cancer of the breast':ab,ti OR 'malignant neoplasm of breast':ab,ti OR 'breast malignant neoplasm':ab,ti OR 'breast malignant neoplasms':ab,ti OR 'malignant tumor of breast':ab,ti OR 'breast malignant tumor':ab,ti OR 'breast malignant tumors':ab,ti OR 'mammary cancer':ab,ti OR 'cancer, mammary':ab,ti OR 'cancers, mammary':ab,ti OR 'mammary cancers':ab,ti OR 'mammary neoplasms, human':ab,ti OR 'human mammary

	neoplasm':ab,ti OR 'human mammary neoplasms':ab,ti OR 'neoplasm, human mammary':ab,ti OR 'neoplasms, human mammary':ab,ti OR 'mammary neoplasm, human':ab,ti OR 'breast carcinoma':ab,ti OR 'breast carcinomas':ab,ti OR 'carcinoma, breast':ab,ti OR 'carcinomas, breast':ab,ti OR 'carcinoma, human mammary':ab,ti OR 'carcinomas, human mammary':ab,ti OR 'human mammary carcinomas':ab,ti OR 'mammary carcinomas, human':ab,ti OR 'human mammary carcinoma':ab,ti<br>#2 curcumin:ab,ti OR curcuma:ab,ti OR 'curcumin phytosome':ab,ti OR 'phytosome, curcumin':ab,ti OR diferuloylmethane:ab,ti OR 'turmeric yellow':ab,ti OR 'yellow, turmeric':ab,ti OR mervia:ab,ti OR curcumas:ab,ti OR 'curcuma longa':ab,ti OR 'curcuma longas':ab,ti OR 'longa, curcuma':ab,ti OR tumeric:ab,ti OR tumerics:ab,ti OR turmeric:ab,ti OR turmerics:ab,ti OR 'curcuma zedoaria':ab,ti OR 'curcuma zedoarias':ab,ti OR 'zedoaria, curcuma':ab,ti OR 'zedoary zedoaria':ab,ti OR 'zedoaria, zedoary':ab,ti OR 'zedoary zedoarias':ab,ti<br>#3 (#1) AND (#2)
