# Supplementary material for: The potential value of curcumin in breast cancer: a systematic review and meta-analysis of preclinical studies
Source: Front Pharmacol. 2026 Jun 24;17:1693452. doi: 10.3389/fphar.2026.1693452 (PMC13341899; doi:10.3389/fphar.2026.1693452)
Supplement: Supplementary file 2 [file Supplementaryfile2.pdf]

Supplementary Material 2：Experimental details in the study

	Study	Country	Animal Model	Control	Intervention	Duration	Outcome
1	A. Grill （2011）	USA	Transgenic mouse model （HER-2+ breast cancer）	Blank PLGA microparticles (n=6)	58.2 mg curcumin-loaded PLGA microparticles（s.c.） (n=6)	84 d	5
2	B. B. Aggarwal （2005）	USA	Female athymic nude mice (MDA-MB-435 xenograft)	Standard diet (n=15)	2.0% curcumin diet (n=15)	35 d	5
3	B. Yadav（2012）	New Zealand	Female CD1 athymic nude mice （MDA-MB-468 xenograft）	water (n=5)	8.5 mg/kg/day RL66 (curcumin analog) (i.g.) (n=5)	70 d	2
4	H. J. Kang（2009）	Korea	Nude mice (MDA-MB-231 xenograft)	water (n=10)	100 mg/kg curcumin (oral) (n=10)	35 d	6
5	J. Zhang （2022）	China	BALB/c mice (4T1 cell-induced tumor)	Saline (n=8)	150 mg/mL Curcumin (oral) (n=8)	28 d	1；2；3；4
6	K. Pal （2019）	India	Female BALB/C mice (4T1 cell-induced tumor)	PLGA (n=5)	PLGA@CCM microspheres （i.p.） (n=5)	20 d	1；2；4；7；9
7	K. Singletary （1998）	USA	Sprague-Dawley rats (DMBA-induced tumors)	Standard diet (n=27)	1.0% curcumin diet (n=25)	14 d	3；4
8	L. E. Wright（2013）	USA	Nude mice (MDA-MB-231 xenograft)	DMSO vehicle (n=7)	50 mg/kg Curcuminoids (i.p.) (n=7)	21 d	3
9	L. J. Aloor（2023）	India	Male Swiss Albino Mice (DLA-induced solid tumors)	Untreated (n=5)	100 µg/kg Curcumin analogue NLH (i.p.) (n=5)	28 days	2
10	L. Lin （2010）	USA	Female athymic nude mice (MDA-MB-231 xenograft)	DMSO vehicle (n=5)	50 mg/kg FLLL32 (curcumin analog) (i.p.) (n=5)	19 d	2
11	M. Inzunza-Soto （2023）	Mexico	Female BALB/c mice (4T1 cell-induced tumor)	Saline (n=5)	5 mg/kg Curcumin (po) (n=5)	21 d	1；2；4
12	M. T. Hsieh（2017）	China	Nude mice (MDA-MB-231 xenograft)	Propylene glycol (vehicle) (n=11)	100 mg/kg/day Curcumin (po) (n=11)	32 d	2
13	N. Abd Razak （2017）	Malaysia	Female BALB/c mice (4T1 cell-induced tumor)	untreated (n=6)	50 mg/kg/day Curcumin(po) (n=6)	28 d	2

14	N. Cesilia Arellano-Rodriguez（2022）	Mexico	Balb/c mice (4T1 cell-induced tumor)	Saline (n=5)	100 mg/k Curcumin (i.p.) (n=5)	21 d	2
15	O. N. K. Martey（2019）	New Zealand	Female SCID mice (MDA-MB-231 xenograft)	SMA micelles (vehicle) (n=8)	SMA-RL71 (curcumin analog RL71 encapsulated in SMA micelles) (i.v.) (n=8)	21 d	5
16	P. Kaya（2019）	Korea	Female MMTV-PyMT transgenic mice (spontaneous mammary tumors and lung metastasis)	Tap water (n=6)	50 mg/kg Curcumae Radix Extract (CRE)(oral) (n=6)	140 d	5
17	P. Tagde（2021）	India	Female Wistar rats (DMBA-induced breast cancer)	Saline (n=6)	40 mg/kg Cur (oral) (n=6)	140 d	1；2；11；12
18	Q. M. Zhou（2009）	China	Female nude mice（MCF-7 xenograft）	Saline (n=8)	100 mg/kg curcumin (i.p.) (n=8)	28 d	1；4；9；10
19	R. Min（2017）	China	Nude mice (MCF-7 xenograft)	IFN-β(s.c.)/RA (n=8)	40μM Curcumin（po）+ IFN-β（s.c.）/RA （po）(halved dose) (n=8)	14 d	6
20	S. Jain（2014）	India	Sprague-Dawley rats (DMBA-induced breast cancer)	untreated (n=8)	10 mg/kg curcumin (i.v.) (n=8)	8 d	7；8
21	S. K. Vemuri（2022）	India	Balb/c mice (4T1 cell-induced tumor)	Saline (n=5)	50 mg/kg Curcumin (i.p.) (n=5)	21 d	2
22	S. Nirgude（2020）	India	Swiss albino mice (EAC cell-induced tumor)	Untreated (n=5)	10 mg/kg ST09 (curcumin derivative)(i.p.) (n=5)	25 d	2；4；7；8；10
23	S. Nirgude（2022）	India	Swiss albino mice (EAC cell-induced tumor)	DMSO vehicle (n=5)	20 mg/kg ST08(20 mg/kg) (i.p.) (n=5)	20 d	4；6；7；8；10；11；12
24	S. Yang（2013）	Korea	Sprague-Dawley rats (DMBA-induced breast cancer)	Saline (n=6)	1000 mg/kg curcuma longa extract (oral) (n=7)	28 d	4；9；10；11；12
25	Somers Edgar（2008）	New Zealand	Female athymic nude mice (MDA-MB-231 xenograft)	5 ml/kg/day Vehicle (n=10)	200 mg/kg/day curcumin(oral) (n=10)	70 d	1；2；7
26	T. C. Cheng（2021）	China	NOD-SCID-IL2Rg^{null} (NSG) mice (patient-derived xenografted tumor)	Vehicle (corn oil) (n=4)	40 mg/kg Curcumin (i.p) (n=4)	12 d	1；2
27	X. Song（2023）	China	Female BALB/c mice (4T1 cell-induced tumor)	NG-PTX (PTX-only) (n=5)	NG-PC (PTX+7.5 mg/kg Cur nanogel) (i.v.) (n=5)	14 d	1；5

28	Y. Duan（2022）	China	BALB/c nude mice (MDA-MB-231 xenograft)	Vehicle (PBS with 6% castor oil) (n=6)	3 mg/kg EF24 (curcumin analog) (i.p.) (n=6)	28 d	2
29	Y. Liao（2024）	China	Female BALB/c mice (4T1 breast cancer)	PBS (n=5)	4 mg/kg Cur (i.v.) (n=5)	16 d	1；2；4
30	Y. Liu（2019）	China	BALB/c nude mice (4T1 cell-induced tumor)	Saline(oral) (n=7)	50 mg/kg CUR (oral) (n=7)	16 d	2；4
31	Y. Zhan（2013）	China	Female Kunming mice (S180 sarcoma cells Intraperitoneal injection)	5 mg/kg Paclitaxel (n=6)	225 mg/kg Curcumin + 5 mg/kg Paclitaxel (i.p.) (n=7)	10 d	1；4
32	Z. D. Lv（2014）	China	Female BALB/c nude mice (MDA-MB-231 xenograft)	Saline (n=8)	200μg/kg Curcumin (i.p.) (n=8)	28 d	1；2
33	Z. Karami（2024）	Iran	Female BALB/c mice (4T1 breast cancer)	Saline (n=6)	30 mg/kg Cur (i.v.) (n=6)	14 d	1；4
